# Comprehensive Optoelectronic Study of Copper Nitride: Dielectric Function and Bandgap Energies

**DOI:** 10.3390/nano15201577

**Published:** 2025-10-16

**Authors:** Manuel Ballester, Almudena P. Marquez, Eduardo Blanco, Jose M. Manuel, Maria I. Rodriguez-Tapiador, Susana M. Fernandez, Florian Willomitzer, Aggelos K. Katsaggelos, Emilio Marquez

**Affiliations:** 1 Department of Computer Sciences, Northwestern University, Evanston, IL 60208, USA; a-katsaggelos@northwestern.edu; 2 Department of Mathematics, University of Cadiz, 11510 Puerto Real, Spain; almudena.marquez@uca.es; 3 Department of Condensed-Matter Physics, University of Cadiz, 11510 Puerto Real, Spain; eduardo.blanco@uca.es (E.B.); jose.manuel@uca.es (J.M.M.); emilio.marquez@uca.es (E.M.); 4 Centre for Energy, Environmental and Technological Research (CIEMAT), Avenida Complutense 40, 28040 Madrid, Spain; mariaisabel.rodriguez@ciemat.es (M.I.R.-T.); susanamaria.fernandez@ciemat.es (S.M.F.); 5 Wyant College of Optical Sciences, University of Arizona, Tucson, AZ 85721, USA; fwillomitzer@arizona.edu; 6 Department of Electrical and Computer Engineering, Northwestern University, Evanston, IL 60208, USA

**Keywords:** copper nitride (Cu_3_N), thin films, ellipsometry, spectrophotometry, optical properties, bandgap analysis, eco-friendly photovoltaic semiconductors

## Abstract

Copper nitride (Cu_3_N) is gaining attention as an eco-friendly thin-film semiconductor in a myriad of applications, including storage devices, microelectronic components, photodetectors, and photovoltaic cells. This work presents a detailed optoelectronic study of Cu_3_N thin films grown by reactive RF-magnetron sputtering under pure N_2_. An overview of the state-of-the-art literature on this material and its potential applications is also provided. The studied films consist of Cu_3_N polycrystals with a cubic anti-ReO_3_ type structure exhibiting a preferential (100) orientation. Their optical properties across the UV-Vis-NIR spectral range were investigated using a combination of multi-angle spectroscopic ellipsometry, broadband transmission, and reflection measurements. Our model employs a stratified geometrical approach, primarily to capture the depth-dependent compositional variations of the Cu_3_N film while also accounting for surface roughness and the underlying glass substrate. The complex dielectric function of the film material is precisely determined through an advanced dispersion model that combines multiple oscillators. By integrating the Tauc–Lorentz, Gaussian, and Drude models, this approach captures the distinct electronic transitions of this polycrystal. This customized optical model allowed us to accurate extract both the indirect (1.83–1.85 eV) and direct (2.38–2.39 eV) bandgaps. Our multifaceted characterization provides one of the most extensive studies of Cu_3_N thin films to date, paving the way for optimized device applications and broader utilization of this promising binary semiconductor, and showing its particular potential for photovoltaic given its adequate bandgap energies for solar applications.

## 1. Introduction

Copper nitride (Cu_3_N) has emerged as a highly promising, earth-abundant, and non-toxic semiconductor for a broad range of applications, including optical storage [1,2,3], microelectronics [4], and photodetection [5]. In the context of photovoltaics, Cu_3_N offers a compelling alternative to crystalline silicon (which currently represents over 95% of the solar cell market) due to its relatively low synthesis temperature, reduced production costs, and potential for tailoring electronic properties through doping [6,7]. Unlike many metastable metal nitride compounds, Cu_3_N remains stable at ambient conditions [8]. These attributes collectively underscore its appeal as a candidate for next-generation solar absorbers in the ongoing pursuit of environmentally benign and scalable energy materials [9,10,11].

**Figure 1:**
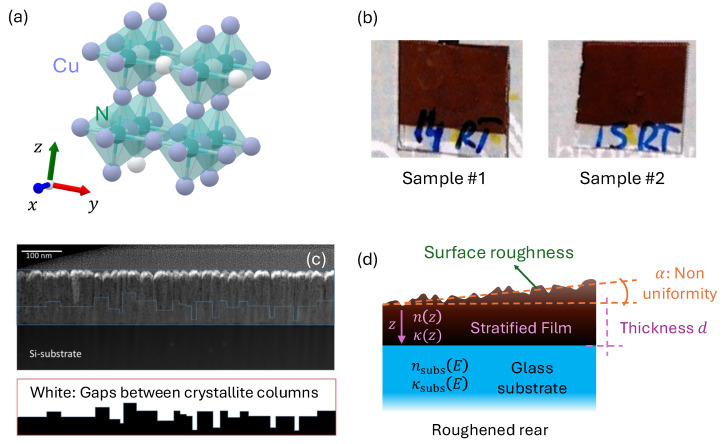
(**a**) Schematic diagram of the cubic Cu_3_N lattice, with nitrogen atoms at octahedral sites and copper at the vertices. (**b**) Photographs of the two representative samples under study. (**c**) Cross-sectional SEM image showing the polycrystalline columns of the thin film. (**d**) Geometrical model for optical analysis.

A defining structural feature of Cu_3_N is its cubic anti-ReO_3_ lattice. Recall that in the ReO_3_ compound, rhenium occupies the corners of an octahedral network, with oxygen at the center of each octahedron. In Cu_3_N, the roles of cations and anions are reversed, placing nitrogen at the octahedral centers while copper atoms occupy the face-centered positions [12], as shown in Figure 1a. Consequently, a large vacant site appears at the body center of the unit cell, rendering the crystal lattice interstitial and allowing deviations from perfect stoichiometry. Such a non-stoichiometric nature endows Cu_3_N with the flexibility to be doped or to accommodate various atomic substitutions, thereby tuning not only its morphology but also its optical and electronic properties [13]. Experimental analyses [14,15] have found that the lattice constant of polycrystalline Cu_3_N typically ranges from approximately 3.815 Å to 3.885 Å, depending on the deposition conditions.

One of the most appealing electronic properties of Cu_3_N is its highly tunable bandgap. The reported experimental optical ranges corresponding to the bandgaps of copper nitride thin films, prepared by different deposition techniques are [16]: (i) direct gaps, approximately 1.2–2.4 eV, and (ii) indirect gaps, approximately 0.6–1.9 eV. On the other hand, standard density functional theory, particularly by local density approximation, predicts a small gap of about 0.9 eV (they systematically and significantly underestimate the electronic bandgap energy of semiconductors and insulators) [17,18]. For photovoltaic applications, semiconductors ideally possess a bandgap in the range of approximately 1.1–1.6 eV, maximizing efficiency for single-junction solar cells under standard solar illumination conditions at the Earth’s surface [19]. In multi-junction architectures, optimal bandgaps typically span a narrower range (e.g., from about 1.7 to 1.9 eV for double-junction devices) depending on the number of sub-cells and whether the design targets diffuse or direct sunlight [19]. The bandgap energy determines the threshold energy for photon absorption, as only photons with energies exceeding the bandgap can generate electron-hole pairs via interband transitions, thus enabling photocurrent generation in photovoltaic devices. Therefore, the broad tunability of the Cu_3_N bandgap offers significant flexibility for optimizing optical absorption and charge carrier generation, making it a promising candidate for use in photovoltaic absorber layers, hypothetically including both homo-junction and hetero-junction designs [20].

While Cu_3_N offers promising advantages, a thorough understanding of the optical and electronic properties remains an ongoing challenge. This difficulty is largely attributed to its metastable nature and the extreme sensitivity of its structural phases to synthesis conditions [21,22,23] and the formation of defects during crystal growth [8]. Reactive radio-frequency magnetron sputtering (RFMS), a prevalent technique for depositing copper nitride films, provides extensive control over key preparation parameters, including substrate temperature, RF power, deposition rate, and chamber pressure. Variations in these parameters, along with the choice between pure N_2_ and N_2_–Ar gas mixtures, have been shown to profoundly impact the crystal stoichiometry, crystallinity, and optical characteristics [10,24]. Therefore, precise tuning of the deposition parameters is essential for tailoring the properties of Cu_3_N to achieve desired film applications.

In this work, we present a comprehensive optoelectronic analysis of Cu_3_N thin films grown by RF magnetron sputtering in a pure N_2_ atmosphere on quasi-transparent substrates. A brief review of the relevant literature and the methodology employed for the analysis of this material is also provided. Two representative samples, prepared under different deposition conditions, are selected to demonstrate our methodology and are shown in Figure 1b. This methodology employs a combination of characterization techniques to achieve a holistic analysis of the film material. Initially, we conduct a structural characterization using grazing-incidence X-ray diffraction. Additional scanning and transmission electron microscopy revealed insights into the morphology of the film and crystal dimensions. An energy-dispersive X-ray spectroscopic analysis also enabled the precise determination of elemental composition. The bulk of the present work is then focused on the optical characterization, considering multi-angle spectroscopic ellipsometric measurements combined with the broadband normal-incidence transmittance and reflectance spectra of the films, capturing both phase-sensitive and intensity-based data. By combining spectroscopic ellipsometry with photometry, we highlight how these complementary approaches enhance the reliability of the extracted optical constants and refine the determination of both direct and indirect bandgaps.

In our proposed optical dispersion model, we parameterize the dielectric function as a function of photon-energy through a set of real coefficients that encapsulate the key features of multiple electronic transitions. Specifically, our customized model comprises a linear combination of multiple oscillators, including the Drude, Tauc–Lorentz, Gaussian, and Wemple–DiDomenico terms. The model parameters provide direct insight into free-carrier dynamics in the NIR region as well as interband transitions extending across the visible and ultraviolet ranges.

Importantly, the cross-sectional images of the thin films (Figure 1c) revealed a pronounced depth-dependent microstructure, with a porous region near the surface gradually transitioning to a denser layer near the substrate. To account for these depth-dependent variations of the density (which can significantly influence the optical response), we introduce, for the first time, a stratified model in which the Cu_3_N film is represented as a series of artificial sub-layers with distinct properties along the depth (*z*-direction), as illustrated in Figure 1d. Last but not least, to clearly highlight the main novel contributions of the present comprehensive and rigorous study, in comparison with previous work on Cu_3_N thin films, it should be emphasized that one relevant innovation lies in the proposed depth-resolved dielectric modeling, another in the devised and practically useful automated Tauc-extrapolation fitting methodology, and, finally, in the systematic comparison of the stoichiometric effects on the optoelectronic properties of copper nitride thin layers presented throughout this work.

## 2. Sample Preparation

Cu_3_N thin films can be synthesized using a wide range of deposition techniques, including chemical vapor deposition (CVD) [25,26] and its plasma-enhanced variants [27,28], atomic layer deposition [29,30], pulsed laser deposition [15,31], molecular beam epitaxy (MBE) [32,33], and physical vapor deposition methods such as reactive DC and RF magnetron sputtering. The first successful deposition of Cu_3_N films using RF sputtering was reported by Terada et al. [34] in 1989. Since then, numerous studies [35,36,37,38] have systematically optimized deposition parameters (such as nitrogen partial pressure, substrate temperature, and sputtering power) to control film stoichiometry, crystal grains, and optoelectronic properties. RF magnetron sputtering arguably became the most widely employed technique for Cu_3_N thin-film growth, owing to its scalability, low processing temperatures, and compatibility with large-area substrates. Gordillo et al. [39] later extended Cu_3_N growth and analyses to reactive DC (triode) sputtering, enabling the fabrication of nitrogen rich films with tunable properties (an approach further explored in [40,41,42]). More recently, alternative techniques such as high-power impulse magnetron sputtering (HiPIMS) [43] have also been investigated, further broadening the range of available synthesis methods for Cu_3_N thin films.

In the present work, we focus on two representative Cu_3_N samples, selected to demonstrate the proposed optoelectronic analysis. Both samples were prepared by the classical reactive RF magnetron sputtering under different conditions and on different substrates. In any case, the presented methodology naturally extended to the analysis of other Cu_3_N films grown with different deposition techniques or preparation conditions. Our first sample was deposited on an n-type Si (100) wafer grown by the floating-zone method, which was pre-polished to remove its native oxide layer. The second sample was grown on a standard commercial glass substrate (Corning 1737F), widely used in optical and display applications. An ultrasonic bath removed the residual contaminants of the glass substrate.

In the RF magnetron sputtering process, a magnet positioned behind the high-purity copper target confines the plasma, facilitating the efficient sputtering of Cu atoms, which react with ionized nitrogen to form the Cu_3_N film on the substrate. The RF power enables stable plasma operation at relatively low pressures, promoting uniform film growth. The depositions were performed using a single-chamber sputtering system (MKSystem LLC). Prior to deposition, the chamber was evacuated to a base pressure of $2.6\times 10^{-5}$ Pa. A 3-inch diameter, 4N purity copper target was used, with a fixed target-to-substrate distance of 10 cm (an important parameter known to vary Cu_3_N film properties [44]). High-purity 5N nitrogen gas was introduced via a mass-flow controller, and the working pressures were set to 3.5 Pa and 5.0 Pa for sample #1 and sample #2, respectively, by adjusting a butterfly valve. All depositions were carried out at room temperature for 30 min using 50 W of RF power at 13.56 MHz.

## 3. Structural Analysis with X-Ray Diffraction

The crystal structure and phase composition of the two representative Cu_3_N films were analyzed using a Malvern PANalytical X’Pert Pro diffractometer, configured in reflection mode with a standard Bragg–Brentano geometry. A Cu K − α radiation source was used to generate the incident X-rays (λ=1.5406 Å), and diffraction data were collected over a 2θ range from 10° to 60°.

**Figure 2:**
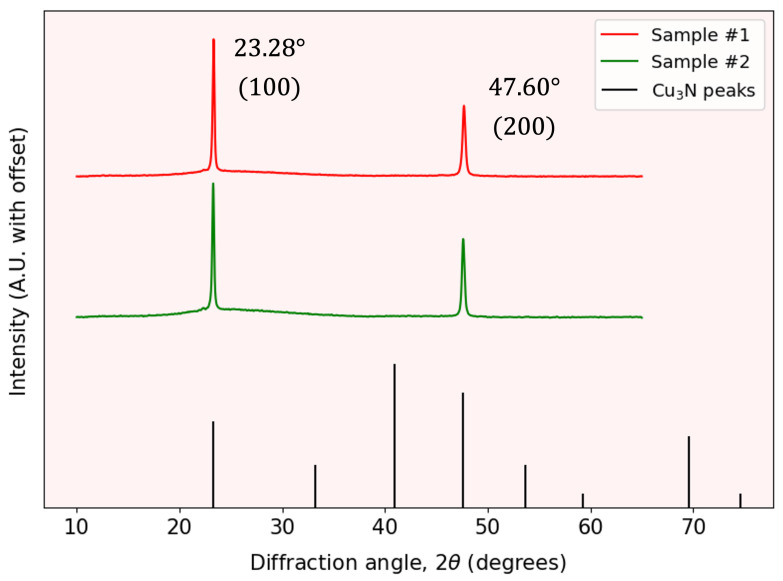
Grazing incidence XRD patterns of representative samples #1 and #2, showing the characteristic peaks corresponding to the cubic lattice.

Figure 2 shows the grazing-incidence XRD (GI-XRD) patterns for representative samples #1 and #2. GI-XRD employs a shallow incidence angle, ensuring the X-rays penetrate only the top few nanometers of the material. This configuration enhances sensitivity to the thin film’s surface structure while minimizing contributions from the underlying substrate. The diffraction patterns reveal prominent peaks at 2θ=23.28° and 47.60°, corresponding to the Cu_3_N lattice planes (100) and (200), respectively. Note that not all of the reflections expected from powder Cu_3_N, as seen at the bottom of the Figure 2, are present in our thin-film patterns. This is a common film XRD feature, since the crystalline domains are not randomly oriented but instead exhibit preferred orientations imposed by the substrate and growth conditions. Only those lattice planes aligned to satisfy the Bragg condition under the chosen geometry contribute detectable peaks. The high intensities and narrow widths of these peaks confirm a well-ordered, polycrystalline structure.

The inter-planar distance $d_{\mathrm{hkl}}$ was determined from Bragg’s law:

(1) $$d_{\mathrm{hkl}}=\frac{\lambda }{2\sin\theta },$$

where λ is the X-ray wavelength and θ the Bragg diffraction angle for each reflection peak. Observe that we have to calculate this distance for the different Miller indices (hkl) for each peak based on the known cubic crystal structure. In particular, the nitrogen atoms located at the 1*a* Wychoff position (0,0,0) and the three copper atoms are located at the 3*d* (1/2,0,0), (0,1/2,0) and (0,0,1/2), leaving a (1/2,1/2,1/2) vacancy. In these type of cubic system, this distance is related to the lattice constant *a* by

(2) $$d_{\mathrm{hkl}}=\frac{a}{h^{2}+k^{2}+l^{2}}.$$

Table 1 presents the lattice constant *a* for both samples. Additionally, the crystallite size *L* was estimated using the Scherrer equation [45]:

(3) $$L=\frac{k\lambda }{\beta \cos\theta },$$

where k≈0.9 is a shape factor and β is the full width at half maximum of the principal peak. The calculated crystallite sizes are also presented in Table 1. These findings demonstrate that the Cu_3_N layers exhibit a high degree of crystallinity and a relatively uniform nano-crystalline domain size, making this semiconductor material promising for applications in photovoltaics.

During the formation of copper nitride thin films, crystalline imperfections are frequently observed and significantly influence their physical properties. The stress generated during film growth can lead to measurable bending of the substrate, reflecting the substantial intrinsic stresses within the coating. In crystalline materials undergoing elastic deformation, changes in lattice spacing reflect the extent of that deformation. In nano-crystalline films, structural defects (including grain boundaries, dislocations, and vacancies) introduce an excess free volume. When this additional free volume interacts with the forces arising from surface and interfacial tensions, it leads to a further increase in the total stress within the thin film.

The lattice distortions were quantified by calculating the micro-strain $\epsilon_{\mathrm{strain}}$ using the following relation:

(4) $$\epsilon_{\mathrm{strain}}=\frac{B}{4\tan\theta },$$

where *B* represents the broadening (in radians) of the main diffraction peak. The $\epsilon_{\mathrm{strain}}$ value reflects how far atoms have been displaced from their ideal lattice positions, which can occur during high-energy growth processes. A lower micro-strain value thus indicates fewer lattice distortions, corresponding to a higher degree of crystallinity in the film. Additionally, a large number of grains with varying orientations contributes to these atomic displacements as the crystalline lattice forms. Table 1 summarizes the micro-strain data obtained from the GI-XRD measurements.

## 4. Morphological Study

### 4.1. Electron Microscopy

Scanning electron microscopy (SEM) employs a finely focused electron beam that rasters across the sample surface. While most primary electrons pass through the specimen without contributing to the image, the secondary electron signal provides topographical contrast with a shallow penetration depth at relatively low accelerating voltages (∼5 kV). At higher accelerating voltages (∼30 kV), high-angle annular dark-field (HAADF) detection can be employed to enhance atomic-number contrast.

On the other hand, transmission electron microscopy (TEM) forms images by transmitting an electron beam through a thin specimen. Conventional direct-beam TEM typically relies on bright- or dark-field modes at lower magnifications. In scanning TEM (STEM), a convergent electron probe is scanned over the sample to form an image, often coupled with HAADF detectors for compositional contrast. Most importantly, high-resolution TEM (HR-TEM) enables phase-contrast imaging at atomic resolution, allowing direct visualization of crystal lattice fringes and defects.

**Figure 3:**
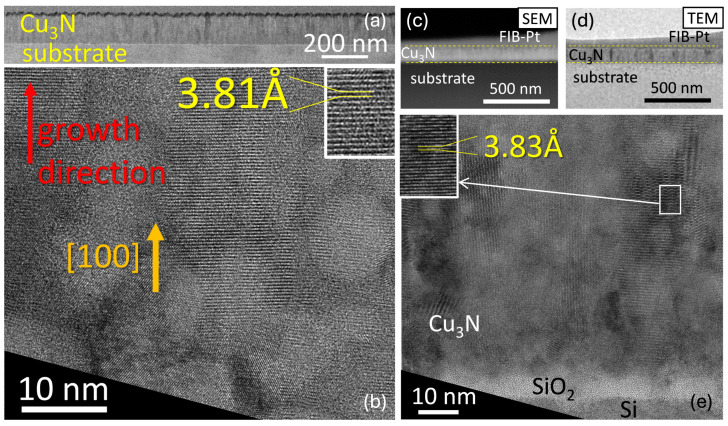
(**a**) Dark-field TEM image (top) from a micrometric region of the material, and (**b**) high-resolution TEM image from Cu_3_N in sample #1. (**c**) high-angle annular dark-field STEM and (**d**) dark-field TEM images of sample #2. (**e**) High-resolution TEM micrograph of Si/SiO_2_/Cu_3_N interfaces.

In this study, SEM observations were performed with a ThermoFisher-Scios2 SEM (Brno, South Moravian Region, Czech Republic) microscope, operating at 5 kV for SE imaging and 30 kV for HAADF. HR-TEM and STEM analyses were carried out on a ThermoFisher STEM instrument (Eindhoven, North Brabant, Netherlands) at 200 kV. Because TEM requires electron-transparent specimens, cross-sectional lamellae of the Cu_3_N thin films were prepared via focused ion beam milling. Prior to milling, protective gold and platinum layers (denoted as Au/BIM-Pt in Figure 3) were deposited to prevent damage from the Ga^+^ ion beam, which was used to carve parallel cuts above and below the region of interest, resulting in a U-shaped trench for sample extraction.

Figure 3a presents a low-magnification dark-field TEM view of the cross section of sample #1, where the columnar morphology is evident from the vertical contrast variations. One can see that overall film thickness remains fairly uniform at the micrometer scale, as quantitatively indicated by SEM measurements of 117.9±8.0 nm and TEM measurements of 123±4 nm. In Figure 3b, the high-resolution TEM image of sample #1 shows voids, with the (100) growth direction again dominating, and we measure the lattice constants to be 3.81 Å for sample #1.

Similarly, the Figure 3c,d compare the cross-sectional high-angle annular dark-field STEM and dark-field TEM images of sample #2, both with identical magnifications, revealing film thicknesses of 109±6 nm (SEM) and 105±5 nm (TEM), in good mutual agreement. The high-resolution TEM image in Figure 3e further highlights the polycrystalline nature of the film, showing nanometer-scale spherical features consistent with small voids, similar to those found in sample #1. Atomic-resolution imaging confirms a preferential (100) orientation perpendicular to the substrate and reveals minimal lattice distortion, as indicated by measured lattice spacings corresponding to Cu_3_N. The lattice constant is 3.83 Å for sample #2, which confirms the reproducible Cu_3_N phase, demonstrating excellent consistency in the deposition process. Overall, SEM and TEM analyses reveal that the Cu_3_N layers have uniform thickness, polycrystalline columnar grains, and a strong (100) preferred orientation. Localized voids do not appear to significantly disturb the crystal structure, as verified by consistent lattice-constant values across these two representative samples.

### 4.2. Crystallographic Analysis with Fourier Optics

The discrete Fourier transform of the high-resolution TEM images, shown in Figure 3b–e, was calculated to obtain reciprocal-space information from the atomic structure. This approach enables the extraction of spatial-frequency data, providing direct insight into the crystallographic ordering. In particular, the three-dimensional representation of the frequency space reveals the Ewald sphere, illustrating how diffracted electron beams **$\vec{k}$** vectors) relate to the polycrystalline structure of Cu_3_N.

**Figure 4:**
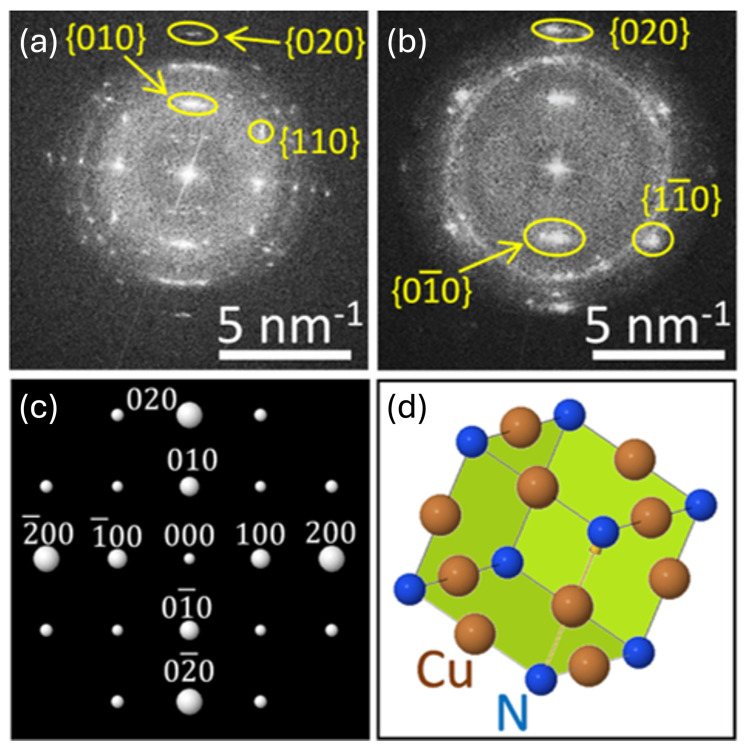
Magnitude of the discrete Fourier transforms of the high-resolution TEM images of (**a**) sample #1 and (**b**) sample #2. (**c**) Simulated electron diffraction pattern with Miller indices for Cu_3_N (001) zone axis. Observe that the indexes of unlabeled peaks correspond to the sum of the orthogonal peaks. (**d**) Simulated unit cell for Cu_3_N.

The resulting Ewald spheres of both samples, shown in Figure 4a,b, depict clear distinct spots consistent with a polycrystalline cubic nitride. When the (001) axis is oriented parallel to the electron beam, reflections from the (100) and (010) lattice planes appear. Due to the multiplicity of the different crystallites, these reflections form arcs or rings rather than discrete points.

To better interpret the observed patterns, simulations were performed using the *Eje-Z* software [46,47,48]. By incorporating lattice constants measured from the high-resolution TEM images, the simulated (001) diffraction pattern (see Figure 4c) confirms the indexing of the experimentally observed peaks. Additional spots arise from overlapping reflections that are characteristic of cubic structures with multiple crystallographic domains. Meanwhile, the simulated Cu_3_N unit cell plotted in Figure 4d highlights the (001) direction (indicated by the yellow arrow) and its correspondence to the experimental geometry, reinforcing the conclusion that the film retains a well-defined cubic lattice orientation.

### 4.3. Composition and Stoichiometry

**Figure 5:**
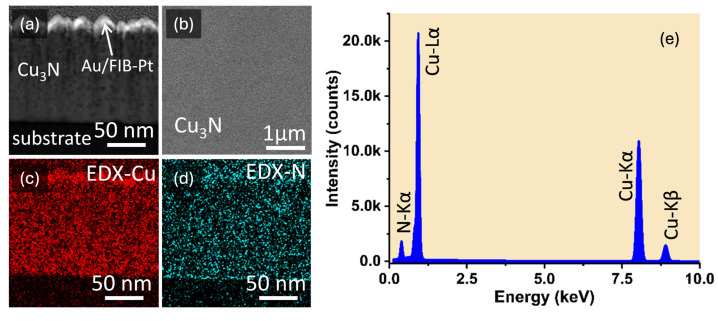
(**a**) Cross-section high-angle annular dark-field STEM and (**b**) top-view of surface SEM images for sample #1. In the same region, the corresponding EDX maps for (**c**) Cu-signal and (**d**) N-signal. (**e**) EDX integrated spectrum from Cu_3_N layer in sample #1.

The SEM/TEM images clearly indicate a homogeneous chemical composition throughout the RF-sputtered Cu_3_N film. Darker regions in cross-sectional images correspond to local voids or minor thickness variations. For instance, the HAADF-STEM cross-sectional image of sample #1 is shown in Figure 3a. A top-view image (top right in that figure) shows the surface roughness, which stems primarily from the columnar growth. The copper and nitrogen elemental maps shown in Figure 5c,d do not reveal any notable deficiencies in either element. While the integrated spectra confirm that sample #2 exhibits a nearly stoichiometric Cu_3_N composition (one nitrogen per three copper atoms), sample #1 is slightly sub-stoichiometric. Figure 3e shows the integrated energy-dispersive X-ray (EDX) spectrum of the Cu_3_N layer for sample #1, where characteristic peaks for Cu and N confirm the composition of the film.

## 5. Spectroscopic Ellipsometry and Photometry

### 5.1. Data Acquisition

**Figure 6:**
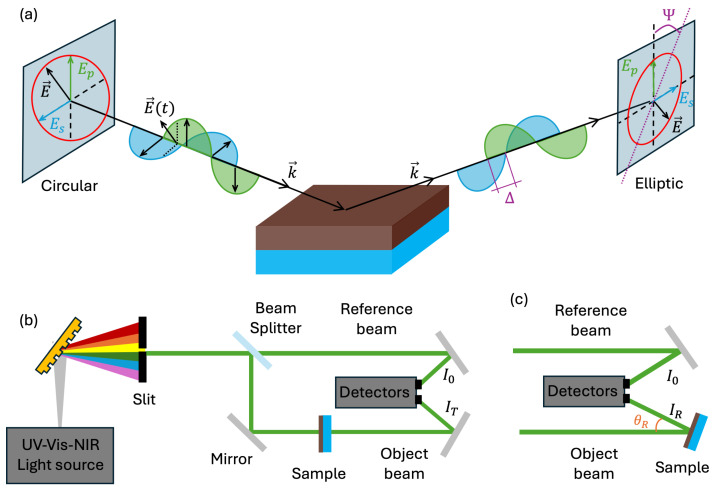
Diagrams of experiments for (**a**) ellipsometric, (**b**) transmittance, and (**c**) reflectance measurements.

Ellipsometry quantifies the amplitude ratio Ψ and phase difference Δ between the *p*- and *s*-polarized components of a light beam specularly reflected from a sample at an incidence angle $\phi_{a}$. A schematic of the experimental configuration is shown in Figure 6a. The measured ellipsometric data (Ψ,Δ) are related to the Fresnel reflection coefficients $r_{p}$ and $r_{s}$ through the complex reflectance ratio ρ [49]:

(5) $$\rho =\tan\left[\Psi (E)\right]\exp\left[i\Delta (E)\right]\equiv \frac{r_{p}}{r_{s}}.$$

For isotropic, non-depolarizing samples, Equation (5) fully describes the system. In practical measurements, however, layer-thickness non-uniformity, surface roughness, or diffuse scattering can introduce a certain level of depolarization. The depolarization factor can be defined as

(6) $$\%\mathrm{depol}=\left(100\%\right)\times \left(1-\sqrt{\alpha^{2}+\beta^{2}+\gamma^{2}}\right),$$

where

(7) α=cos(2Ψ),β=sin(2Ψ)cos(Δ),andγ=sin(2Ψ)sin(Δ).

Once the depolarization is calculated by using an autoretarder, this factor can be correlated with the geometry of a non-ideal sample, and we can then determine a non-uniformity thickness parameter denoted by $N_{d}$.

In this work, we used a commercial spectroscopic ellipsometer (VASE, J.A. Woollam Co. Inc. [Lincoln, NE, USA]) to examine a set of two Cu_3_N thin films. The ellipsometric parameters Ψ and Δ were measured at room temperature from 250 to 2500 nm (0.50–4.96 eV) with a 10 nm spectral resolution and at three incidence angles (50°, 60°, and 70°), which are near the Brewster angle for these samples.

In the ellipsometer, the incident beam is first conditioned with a Berek computer-controlled adjustable compensator, introducing a precise phase shift δ∈[0,90°] between the *s*- and *p*- polarized wave components. This phase shift transforms the initial linearly polarized input beam into any other desired polarization state (represented for visual purposes as circular in Figure 6, considering the case of δ=90°), which enhances the sensitivity of the measurement. Upon reflection from the thin film, the polarization state of the light becomes elliptical, reflecting the optical properties of the sample [50]. A rotating analyzer in the detection path modulates the reflected beam, enabling the extraction of the normalized Fresnel reflection coefficients. These coefficients, which quantify the amplitude and phase changes of both the *s*- and *p*-components, are then converted into the ellipsometric raw data Ψ and Δ. Using a corresponding dispersion model, these data can be fitted to a theoretical model using the WVASE (v3.9.42) software, ultimately yielding precise determinations of the thickness and complex dielectric function of the film.

Ellipsometry offers phase-sensitive data over a broad spectral range and different incidence angles, enabling accurate determination of the optical functions. Nevertheless, direct intensity measurements (transmittance and reflectance) using unpolarized light are less influenced by surface topology and depolarization effects, providing complementary information. Accordingly, we also acquired normal-incidence transmittance data (Figure 6b) and near-normal-incidence ($\theta_{R}=6{}^{\circ}$) reflectance data (Figure 6c), using a PerkinElmer Lambda-1050 double-beam UV-Vis-NIR (Waltham, MA, USA) spectrophotometer with UV WinLab (v7.5) software. The double-beam setup employs separate detectors for a sample and reference beams, thus compensating for rapid fluctuations in the light source and yielding highly reliable measurements.

### 5.2. Optical Functions

Within the framework of Maxwell’s equations for non-magnetic materials, the complex refractive index N(E)=n(E)+ik(E) is related to the complex dielectric function $\epsilon \left(E\right)=\epsilon_{1}\left(E\right)+i\epsilon_{2}\left(E\right)$ by the simple expression $N^{2}=\epsilon$. Hence,

(8) $$\begin{array}{cc} \epsilon_{1} & =n^{2}-k^{2}, \end{array}$$

(9) $$\begin{array}{cc} \epsilon_{2} & =2nk. \end{array}$$

If the extinction coefficient k≈0, it follows that $\epsilon_{2}\approx 0$ and $\epsilon_{1}\approx n^{2}$, illustrating a clear linear relation between $\epsilon_{2}$ and the material’s absorption features. Conversely, given $\left(\epsilon_{1},\epsilon_{2}\right)$, the quantities (n,k) can be retrieved via

(10) $$\begin{array}{c} n=\sqrt{\frac{\epsilon_{1}+\sqrt{\epsilon_{1}^{2}+\epsilon_{2}^{2}}}{2}}, \end{array}$$

(11) $$\begin{array}{c} k=\sqrt{\frac{-\epsilon_{1}+\sqrt{\epsilon_{1}^{2}+\epsilon_{2}^{2}}}{2}}. \end{array}$$

From *k*, one obtains the absorption coefficient as $\alpha =\frac{4\pi k}{\lambda }$. This parameter governs the decay of light intensity upon traversing a layer of thickness *d*. Specifically, for a collimated beam with intensity $I_{0}$, the Beer–Lambert law gives $I_{T}=I_{0}e^{-d\alpha }$ for a single pass transmission without back reflections.

In this study, we developed a mathematical model to describe the complex optical functions, N(E) or ϵ(E), via a set of real-valued parameters $\Theta \in R^{k}$. We express the dispersion as a linear combination of multiple oscillators, each associated with particular electronic transitions of the thin-film material. Details of this dispersion model are discussed extensively in the following section.

We further assume a multilayer (stratified) geometric model in which the thin film is artificially subdivided into several homogeneous sub-layers, each with potentially different optical properties. Figure 1c presents a cross-sectional SEM image of the sample, indicating a voided region near the top (∼36% of the layer thickness) and a denser region (∼64% of the layer thickness) below. Figure 1d schematically shows the corresponding optical model, where each sub-layer can exhibit different (n,k) values. The number of unknown optical parameters thus grows exponentially with the number of layers *l*, so that $\Theta \in R^{k\times l}$. We impose a monotonic decrease in the optical density (both *n* and *k*) from the substrate toward the film surface.

Observe that, although the spectral measurements are taken at equidistance wavelength, the dispersion models are often written in terms of photon-energies. Therefore, for convenience, we will denote the theoretical or simulated optical function as N(λ;Θ) and N(E;Θ) interchangeably, referring to the wavelength or photon energy domain.

### 5.3. Optimization Problem

The WVASE software incorporates a simulator capable of generating transmittance, reflectance, and ellipsometric spectra for given optical parameters Θ, film thickness *d*, and a small thickness non-uniformity $N_{d}$, which is consider as a first order Taylor approximation as a planar wedge. The theoretical transmittance and reflectance are computed through the transfer-matrix formalism, as explained in [51]. These two spectro-photometric datasets clearly capture the Fabry–Perot interference effects, therefore providing a precise determination of *d*. The ellipsometric signals (Ψ,Δ) are simulated by numerically propagating the vector electric field via Jones matrices, thus incorporating polarization effects.

Our aim is to find the optimal parameters $\left(\Theta ,d,N_{d}\right)$ that simultaneously minimize the discrepancy between all the modeled simulated data $\left(T^{\mathrm{mod}},R^{\mathrm{mod}},\Psi^{\mathrm{mod}},\Delta^{\mathrm{mod}}\right)$ and experimental $\left(T^{\exp},R^{\exp},\Psi^{\exp},\Delta^{\exp}\right)$ data. Specifically, we solve a nonlinear optimization problem by minimizing a global mean-squared-error (MSE) metric.

First, the intensity-sensitive errors are defined for transmittance and reflectance as

(12) $$\begin{array}{c} {\mathrm{MSE}}_{T}=\frac{1}{N}\sum_{i=1}^{N}{\left(T_{i}^{\mathrm{mod}}-T_{i}^{\exp}\right)}^{2}, \end{array}$$

(13) $$\begin{array}{c} {\mathrm{MSE}}_{R}=\frac{1}{N}\sum_{i=1}^{N}{\left(R_{i}^{\mathrm{mod}}-R_{i}^{\exp}\right)}^{2}, \end{array}$$

where *N* is the number of measurement points. For the ellipsometric parameters, an error function that incorporates both Ψ and Δ is commonly used, that is,

(14) $$\begin{array}{cc} \; & {\mathrm{MSE}}_{\Psi ,\Delta }=\sqrt{\frac{1}{2N-M}\chi^{2}}, \end{array}$$

(15) $$\begin{array}{cc} \; & \chi^{2}=\sum_{i=1}^{N}\left[{\left(\frac{\Psi_{i}^{\mathrm{mod}}-\Psi_{i}^{\exp}}{\sigma_{\Psi ,i}^{\exp}}\right)}^{2}+{\left(\frac{\Delta_{i}^{\mathrm{mod}}-\Delta_{i}^{\exp}}{\sigma_{\Delta ,i}^{\exp}}\right)}^{2}\right], \end{array}$$

where the experimental standard deviations for Ψ and Δ is denoted by $\sigma_{\Psi }$ and $\sigma_{\Delta }$, respectively. This weighting ensures that the ellipsometric residuals are dimensionless and appropriately normalized.

A Levenberg–Marquardt optimization routine iteratively adjusts Θ, *d*, and $N_{d}$ to minimize the following error function:

(16) $$\begin{array}{c} \arg\min_{\Theta ,d,N_{d}}{\mathrm{MSE}}_{S}\left(\Theta ,d,N_{d}\right), \end{array}$$

(17) $$\begin{array}{c} {\mathrm{MSE}}_{S}=\sqrt{{\mathrm{MSE}}_{\Psi ,\Delta }^{2}+{\mathrm{MSE}}_{T}^{2}+{\mathrm{MSE}}_{R}^{2}}. \end{array}$$

**Figure 7:**
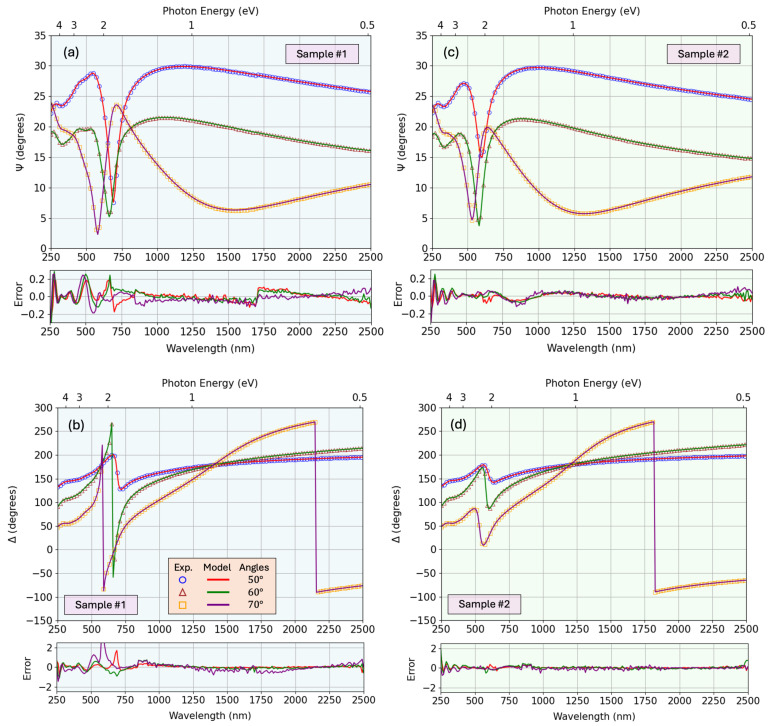
Fitting of Ψ (**top**) and Δ (**bottom**) ellipsometric data for samples #1 (**left**) and #2 (**right**), with the corresponding residual error.

**Figure 8:**
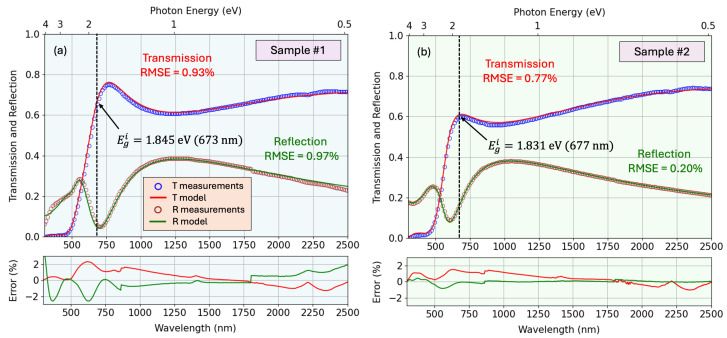
Fitting of *T* and *R* spectro-photometric data for samples #1 and #2, with the corresponding residual error. In this figure are shown both the experimental spectrophotometric data and the simulated curves from our corresponding optical model. The values of the bandgap of the copper nitride samples under study (which will be calculated below), are also indicated.

The resulting best-fit parameters, denoted by $\left(\Theta^{*},d^{*},N_{d}^{*}\right)$, thus yield the simulated spectra that most accurately reflect the experimental data. This multivariate approach leverages the combined sensitivity of ellipsometric and photometric measurements, enabling robust extraction of both optical constants and layer thicknesses. Figure 7 shows the fit of Ψ and Δ for the two representative samples, along with the residual error between the real and modeled curves. Similarly, Figure 8 shows the fit for the transmission and reflection spectra. An interesting counter-intuitive event occurs when the transmission of the full sample is above the transmission of the substrate alone. This happens when the thin film acts as an anti-reflective coating, having a gradient of optical density throughout the depth, with a relatively low refractive index on the upper artificial layer and higher value near the substrate, therefore avoiding pronounced air-film interface that lowers the reflection, and therefore favors the transmission of the beam throughout the sample. The resulting parameters obtained from the fitting process (including the film thickness, non-uniformity, surface roughness, offset term of the real dielectric function, mean-square error of the fit, and the percentage variation of the refractive index with depth) are summarized in Table 2. For comparison, the thickness values determined from profilometry and SEM measurements are also included.

### 5.4. Data Inversion and Pseudo-Dielectric Function

In the next section, we will discuss the accurately calculated dielectric function from the ellipsometric and photometric spectra using our particular optical model. Nevertheless, it should be noted that there is a simple direct inversion of the measured ellipsometry data that yields the complex pseudo-dielectric function 〈ϵ〉. The angled brackets indicate that this quantity represents an effective dielectric response calculated under the simplifying assumptions of a homogeneous, isotropic, and smooth layer. Its real part, $\langle \epsilon_{1}\rangle$, and imaginary part, $\langle \epsilon_{2}\rangle$, can be written (for a sample measured at angle of incidence $\phi_{a}$) as

(18) $$\langle \epsilon \rangle =\langle \epsilon_{1}\rangle +i\langle \epsilon_{2}\rangle =\sin^{2}\left(\phi_{a}\right)\left[1+\frac{{\left(1-\rho \right)}^{2}}{{\left(1+\rho \right)}^{2}}\tan^{2}\left(\phi_{a}\right)\right],$$

where $\phi_{a}$ represents the angle of incidence. Owing to surface roughness and depth-dependent inhomogeneity, the directly inverted values do not correspond to the intrinsic dielectric response of the material. We therefore refer to them as pseudo-dielectric functions, to emphasize their distinction from the true optical functions.

**Figure 9:**
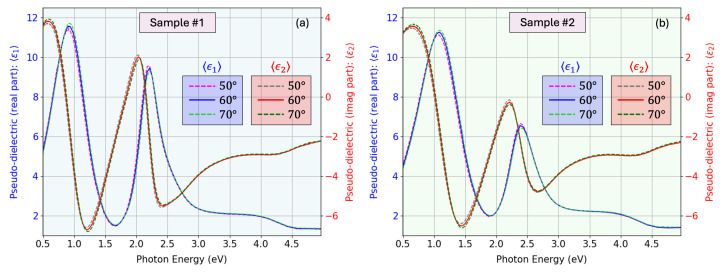
(**a**) Pseudo-dielectric function of sample #2 obtained from the experimental ellipsometric data at 50° and 70° *versus* the pseudo-dielectric function obtained from the recovered fitted curves, showing an excellent match. (**b**) Pseudo-dielectric function of sample #2 obtained at 50°, 60°, and 70°. The observed discrepancies between angles (although the function should ideally be angle independent) demonstrate that a simple homogeneous-film inversion is insufficient and justify the need for the advanced, depth-dependent optical model adopted in this work.

The determination of pseudo-dielectric functions inherently assumes that the film is isotropic and completely homogeneous. The clear angle dependence observed in Figure 9 demonstrates that this approximation is not valid for our samples. To properly capture the optical response, we therefore employ the proposed sophisticated multilayer model in which a number of artificial sub-layers represent the gradual depth-dependent variation in optical density, from the porous surface region with void inclusions to the denser material closer to the substrate. The surface roughness is modeled using the Bruggeman effective medium approximation (BEMA) [52], with a 50:50 mixture of voids and material across the roughness amplitude Rq.

## 6. Optical Dispersion Model

The optical model required to accurately reproduce the experimental data is composed of four physical layers: the glass substrate, the Cu_3_N thin film, a surface roughness layer, and ambient air. Accounting for the surface roughness of the Cu_3_N film significantly improved the match between the experimental and model-generated ellipsometric spectra, making its inclusion in the optical model essential for achieving accurate results. In order to accurately determine the optical properties of the glass substrate (prior to the deposition of the copper nitride film), a standard optical model of the dielectric function of the substrate was employed. In particular, the parameterized dispersion model for the glass employs a combination of oscillators with Gaussian broadening and Sellmeier poles, which ensures Kramers–Kronig (KK) consistency. In other words, we take into account the causality principle in the response of solid materials to an external electric field, related to the fact that the two components of the complex dielectric functions are Hilbert transforms of each other (that is, the well known Kramers–Kronig bidirectional relations).

A more advanced parameterized dispersion model was employed to account for the Cu_3_N thin-film layer. To match the experimental ellipsometric data with our model, this must provide sufficient flexibility for adequate reproduction of the lineshape of the bandgap and other higher-order electronic transitions. In Figure 7, a comparison of the experimental and model-generated ellipsometric angles Ψ and Δ is shown. It must be emphasized that these parameterized models are advantageous over the so-called “point-by-point” approaches, as they impose the KK-consistency. The point-by-point methods determine the real and imaginary parts of the complex dielectric function, $\epsilon_{1}\left(E\right)$ and $\epsilon_{2}\left(E\right)$, respectively, independently at each measured photon energy *E*. As a result, the complex dielectric function $\epsilon =\epsilon_{1}+i\epsilon_{2}$ may then not satisfy the KK relationship. The use of parameterized optical functions helps prevent measurement noise from being mistakenly incorporated into the complex optical function by avoiding overfitting the experimental data.

The Tauc–Lorentz oscillator dispersion model, originally proposed by Jellison and Modine [53], is usually employed to parameterize the complex dielectric function of amorphous semiconductors. Additionally, Synowicki and Tiwald [54] have demonstrated that complex dielectric function models composed of multiple oscillator types can provide a very flexible approach for analyzing not only amorphous but also crystalline semiconductors. Specifically, the parameterized dielectric function ϵ(E) used in this work to describe the optical response of the copper nitride thin film in the accessible spectral range consists of two oscillators with Tauc–Lorentz broadening, two oscillators with Gaussian broadening, and a contribution from free charge carriers, modeled by the classical Drude model. We then write the mathematical expression for the dielectric function as follows:

(19) $$\begin{array}{ccc} \epsilon (E) & = & \epsilon_{\infty }+\mathrm{Sell}\left(E;A,E_{0}\right)+\sum_{i=1}^{2}{\mathrm{TL}}_{i}\left(E;A,E_{0},\Gamma ,E_{g}^{\mathrm{TL}}\right)\\ & & \;+\sum_{i=1}^{2}{\mathrm{Gau}}_{i}\left(E;A,E_{0},\Gamma \right)+\mathrm{Dru}\left(E;A,\Gamma \right), \end{array}$$

where

(20) $$\begin{array}{ccc} \mathrm{Sell}(E;A,E_{0}) & = & \frac{A}{E_{0}^{2}-E^{2}}, \end{array}$$

(21) $$\begin{array}{ccc} \mathrm{and}\;\mathrm{Dru}(E;A,\Gamma ) & = & -\;\frac{A}{E^{2}+i\;\Gamma \;E}. \end{array}$$

The functions ${\mathrm{TL}}_{i}\left(E;A,E_{0},\Gamma ,E_{g}^{\mathrm{TL}}\right)$, ${\mathrm{Gau}}_{i}\left(E;A,E_{0},\Gamma \right)$ and Dru(E;A,Γ) represent the Tauc–Lorentz, Gaussian, and Drude oscillators, respectively. These functions contribute to the dielectric function by accounting for the electronic bandgap, higher-order electronic transitions, and the free carrier response, as mentioned earlier. The parameters $A,E_{0},\Gamma$, and $E_{g}^{\mathrm{TL}}$ indicate the oscillator amplitude, resonant energy, broadening, and Tauc–Lorentz bandgap energy, respectively.

Contributions from higher energy transitions outside the measured spectral range only affect the dispersion of the real part of the dielectric function, $\epsilon_{1}$. They are incorporated into our model through a Sellmeier pole $\mathrm{Sell}(E;A,E_{0})$ (which is a Lorentz oscillator with negligible broadening) and the constant offset $\epsilon_{\infty }$.

The imaginary component of the dielectric function for the Tauc–Lorentz and Gaussian broadening models, denoted as $\epsilon_{2}^{\mathrm{TL}}$ and $\epsilon_{2}^{\mathrm{Gau}}$, respectively, is expressed analytically as

(22) $$\epsilon_{2}^{\mathrm{TL}}\left(E\right)=\left\{\begin{array}{cc} \frac{AE_{0}\Gamma {\left(E-E_{g}^{\mathrm{TL}}\right)}^{2}}{{\left(E^{2}-E_{0}^{2}\right)}^{2}+\Gamma^{2}E^{2}}\;\frac{1}{E}, & E>E_{g}^{\mathrm{TL}},\\ 0, & E\leq E_{g}^{\mathrm{TL}}. \end{array}\right.$$

(23) $$\epsilon_{2}^{\mathrm{Gau}}\left(E\right)=A\exp\left[-{\left(\frac{E-E_{0}}{f\Gamma }\right)}^{2}\right]-A\exp\left[-{\left(\frac{E+E_{0}}{f\Gamma }\right)}^{2}\right].$$

Here, the constant $f=1/2\sqrt{\ln(2)}$ in Equation (23) defines the full-width half maximum (FWHM) of the Gaussian broadening Γ. The real part of the Tauc–Lorentz and Gaussian dielectric function, $\epsilon_{1}^{\mathrm{TL}}\left(E\right)$ and $\epsilon_{1}^{\mathrm{Gau}}\left(E\right)$, is obtained via the Kramers–Kronig (KK) transformation of their corresponding imaginary components. For instance, the real part of the Gaussian oscillator is given by

(24) $$\begin{array}{c} \epsilon_{1}^{\mathrm{Gau}}\left(E\right)=\frac{2}{\pi }P\int_{0}^{\infty }\frac{\xi \;\epsilon_{2}^{\mathrm{Gau}}\left(E\right)}{\xi^{2}-E^{2}}\;d\xi =\frac{2A}{\sqrt{\pi }}\left[F\left(\frac{E+E_{0}}{f\Gamma }\right)-F\left(\frac{E-E_{0}}{f\Gamma }\right)\right], \end{array}$$

where P denotes the Cauchy principal value of the integral and *F* is the Dawson function, which is implemented in the Python (v3.13.9) package SciPy (v1.15.2) [55]. The exact analytical solution for $\epsilon_{1}$ for the Tauc–Lorentz oscillator is provided in [56]. For the Gaussian oscillator, the analytical solution written in Equation (24) was originally derived in [57].

The optical properties of Cu_3_N layers were modeled, to a great extent, using the Tauc–Lorentz equation, which has been demonstrated to be applicable to both amorphous high ˘k oxides (one Tauc–Lorentz oscillator), as well as to polycrystalline oxide films, using in this latter case two Tauc–Lorentz oscillators. The additional oscillator for the case of poly-crystalline layers is used to take into account any singularities in the interband density of states caused by the presence of long-range order in polycrystalline films. In our copper nitride layer under study, the first Tauc–Lorentz oscillator mainly defines the position of the optical bandgap, whereas the second one describes the existing optical absorption above the absorption edge.

**Figure 10:**
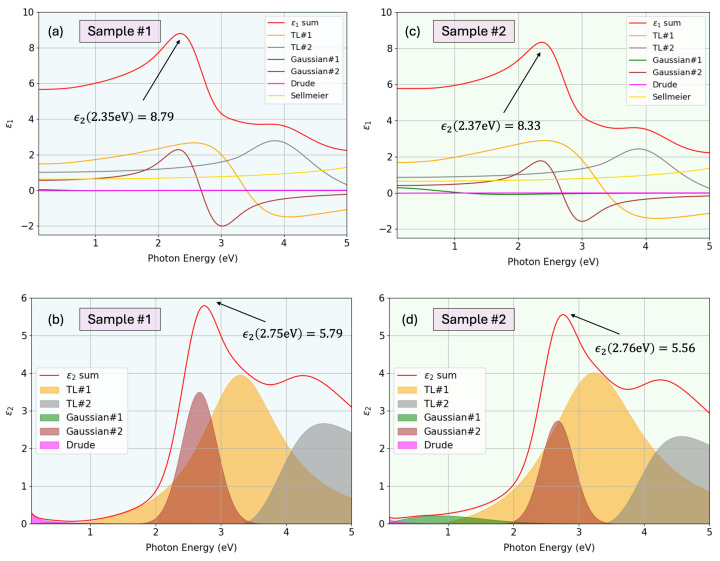
Complex dielectric functions for sample #1 with the different contributions of the oscillators for the (**a**) real and (**b**) imaginary parts, and for sample #2 with (**c**) real and (**d**) imaginary parts.

**Figure 11:**
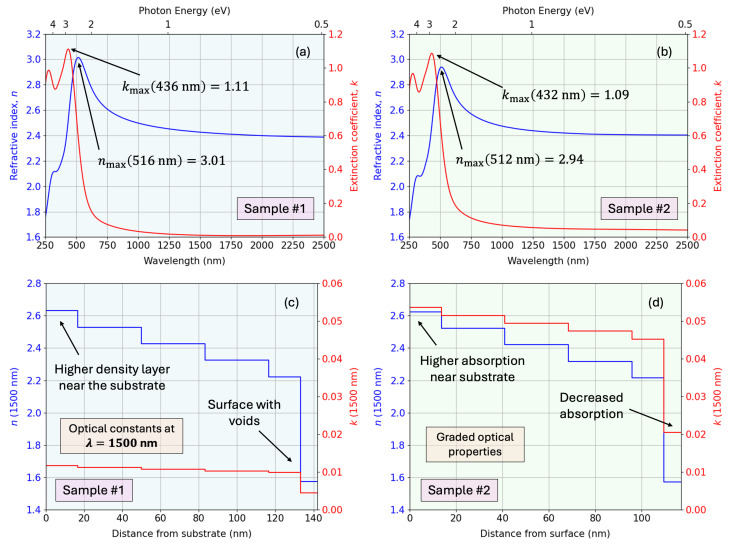
Complex refractive index for (**a**) sample #1 and (**b**) sample #2, and the corresponding depth profile at λ=1500 nm for (**c**) sample #1 and (**d**) sample #2.

Figure 10 shows the calculated complex dielectric functions (real and imaginary components), along with the deconvolution into the individual oscillator contributions. One can find the corresponding found parameters of each oscillator in Table 3. Additionally, Figure 11 presents the corresponding refractive index and extinction coefficient spectra. The figure also depicts the depth-resolved optical constants at λ=1500 nm, illustrating a graded optical structure with higher density and absorption near the substrate and a gradual decrease toward the surface, consistent with the modeled depth-dependent dielectric response.

Overall, it should be finally mentioned that the correlation between the fit parameters has indicated that the optical model adopted in this analysis, with the multiple oscillator models, is unique. The correlation matrices of the parameters have demonstrated that any two parameters are independent: the values of the correlation matrix find values are less than 0.92, which is considered acceptably uncorrelated [58,59]. This means that no more than necessary oscillators were introduced or that there is no redundant information extract (which would lead to ambiguous and non-unique results).

## 7. Drude Model and Optical Resistivity

It must be pointed out that, interestingly, the previous formula in Equation (21) can also be written in terms of the free carrier optical parameters, the so-called (optical) resistivity $\rho_{\mathrm{opt}}$ (units are Ωcm) and the mean scattering time, τ. Observe that “optical” resistivity refers to the conventional electrical resistivity, but when found by precise optical measurements rather than traditional electrical ones. The oscillator can then be expressed as

(25) $$\mathrm{Dru}\left(E;\rho_{\mathrm{opt}},\tau \right)=-\frac{\hbar^{2}}{\epsilon_{0}\rho_{\mathrm{opt}}\left(\tau E^{2}+i\hbar E\right)},$$

where *ℏ* is the reduced Planck’s constant and $\epsilon_{0}$ is the vacuum permittivity constant. Other related parameters of interest include the (optical) carrier concentration, $N_{\mathrm{opt}}$ and the (optical) carrier mobility $\mu_{\mathrm{opt}}$, which can be calculated by

(26) $$\rho_{\mathrm{opt}}=\frac{m^{*}}{N_{\mathrm{opt}}q^{2}\tau }=\frac{1}{q\mu_{\mathrm{opt}}N_{\mathrm{opt}}},$$

where *q* is the single electron charge, and the effective mass $m^{*}$ is given by

(27) $$E\left(k\right)=E_{0}+\frac{{\left(\hbar k\right)}^{2}}{2m^{*}},$$

with E(k) denoting the energy of an electron with wave number *k* and $E_{0}$ the constant band energy. It must also be noted that, in the most general case, the effective mass is defined from the curvature of the electronic band structure as

(28) $$\frac{1}{m^{*}}=\frac{1}{\hbar^{2}}\frac{d^{2}E\left(k\right)}{dk^{2}},$$

which reduces to Equation (27) for a parabolic band dispersion.

Regardless of which expression for the Drude model is chosen (Equation (21) or Equation (25)), it has only two free parameters that describe a shape-line with increasing absorption towards lower photon energies. The carrier concentration and mobility can be determined from the Drude parameters $\rho_{\mathrm{opt}}$ and τ if the effective mass $m^{*}$ is known *a priori*. Thus, considering that our Cu-N samples are slightly N-rich, and taking into account the results by Gordillo et al. [60,61] that these N-rich Cu-N layers show a negative Seebeck coefficient, it is deduced that the majority of free charge carriers have to be electrons. In the stoichiometric Cu_3_N films, the electron and hole carrier densities would be practically compensated, due to their very small value of the Seebeck coefficient. Hence, we have considered the value of the electron effective mass, $m^{*}=0.16m_{0}$ [26], where $m_{0}$ is the free-electron mass. This assumption finally gives rise to an approximate value for $N_{\mathrm{opt}}$ of $6.2\times 10^{18}\;{\mathrm{cm}}^{-3}$ for sample #1 and $1.9\times 10^{18}\;{\mathrm{cm}}^{-3}$ for sample #2. We also found a charge mobility of $\mu_{\mathrm{opt}}=3.947$ and $\mu_{\mathrm{opt}}=6.0$ cm^2^/(Vs), for the two representative Cu-N samples, respectively.

The electrical properties can be determined by optical as well as the conventional electrical van der Pauw method. The results obtained by the two approaches show a certain disagreement in the Cu_3_N literature. A possible explanation can be that both ellipsometry and spectrophotometry are indirect techniques, which require data modeling of the dielectric function, while the electrical parameters are obtained by direct Hall-transport measurements. However, despite the inherent differences, we have found experimentally comparable values for the two properties of interest. The charge-carrier concentration and mobility were determined from Hall-effect measurements performed using an Ecopia HMS-5000 system (Ecopia Corp., Anyang, Republic of Korea) in the van der Pauw configuration, employing suitable aluminum electrodes. The obtained majority charge-carrier mobility of 1.0–3.0 cm^2^V^−1^s^−1^ and the carrier concentration of approximately $1.0\times 10^{17}$ cm^−3^ are reasonably close to the values derived from the Drude model. This agreement illustrates the consistency and reliability of the optical and electrical approaches used in our investigation.

Concerning the electrical resistivity ($\rho_{\mathrm{elect}}$), as measured by the van der Pauw (four-point geometry) method at room temperature, Gordillo et al. [60] have reported an increase of approximately four orders of magnitude, from approximately $10^{-2}\;\Omega \mathrm{cm}$ to nearly $10^{2}\;\Omega \mathrm{cm}$, by varying the nitrogen concentration in copper nitride. On the other hand, the values of the optical resistivity we obtained are very consistent with those reported by Gordillo et al. [60] when using the Hall-transport measurements for the nitrogen concentrations of our RF-sputtered Cu_3_N specimens. Particularly, they measured a range of resistivity values from $10^{-2}\;\Omega \mathrm{cm}$, corresponding to a nitrogen concentration of nearly 28.7%, as compared to the value of 0.52Ωcm we obtained with our optical measurements. It has also been reported by Chen et al. [62], a relatively close value for the Hall-effect electrical resistivity of 1.5Ωcm, although the Cu_3_N material was instead a p-type semiconductor. The good agreement of the found $\rho_{\mathrm{opt}}$ values we obtained with previous reported values of both $\rho_{\mathrm{opt}}$ and $\rho_{\mathrm{elect}}$ supports the validity of our proposed approach.

## 8. Calculations of the Indirect and Direct Bandgap Energies

Generally speaking, each semiconductor material has both an indirect and a direct bandgap. Whichever is lower ultimately defines the nature of its particular fundamental (absolute) bandgap. This bandgap, on the other hand, is a property associated with the corresponding lattice constant. This lattice constant can be changed by various means (e.g., by applying pressure, by heating or cooling, or even by mixing another semiconductor). Both the indirect and direct bandgaps will change with the lattice constant, but at different rates. So, there is a possibility that, at a certain value of the lattice constant, the indirect bandgap could become higher than the direct bandgap. In that case, the semiconductor will behave as a direct-gap semiconductor.

Regarding the electronic band structure properties of Cu_3_N, we now consider its absorption coefficient, α(E), at energies around the absorption edge. It is mentioned that the Brillouin zone locations of the anti-bonding valence-band maximum (high-symmetry R-point), and the bonding conduction-band minimum (high-symmetry M-point), both belonging to the **$\vec{k}$**-reciprocal space, determine the indirect (lowest) R→M gap of Cu_3_N. The M and R, direct energy bandgaps of this copper nitride semiconductor compound need also be considered.

**Figure 12:**
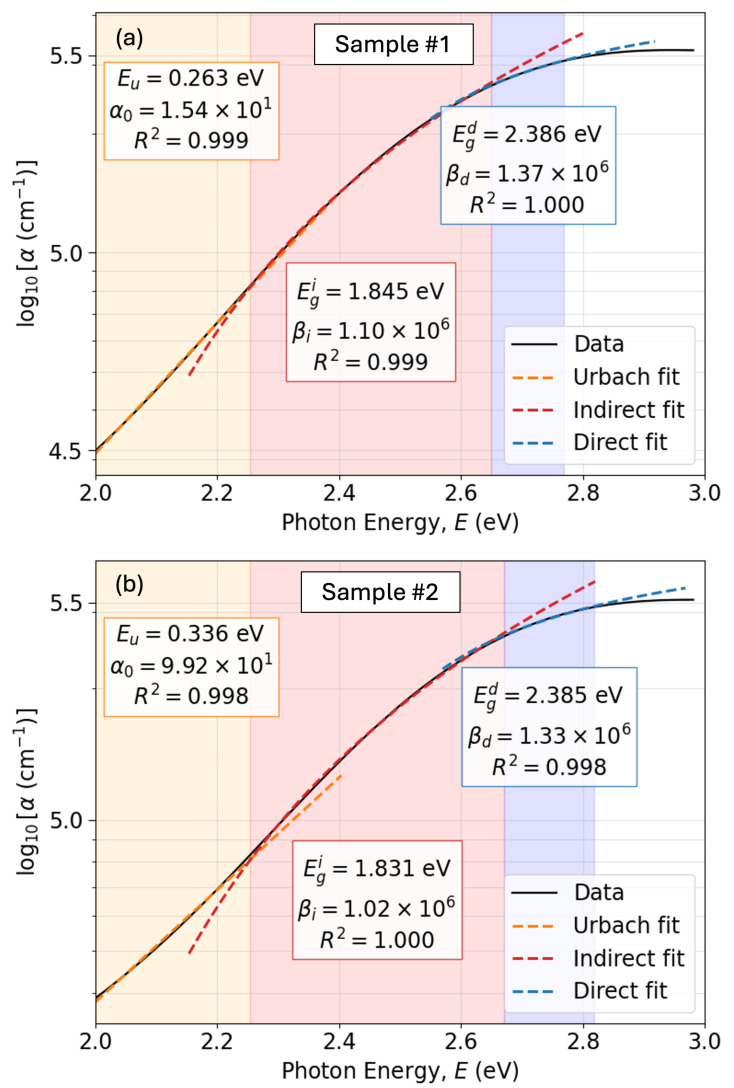
Absorption model for (**a**) sample #1 and (**b**) sample #2. Urbach, indirect, and direct regions are fitted according to the phenomenological model. Note: Units are omitted in the plots for clarity. Photon energy and bandgap energies are in eV, $\alpha_{0}$ in cm^−1^, $\beta_{i}$ in cm^−1^ eV^−1^, and $\beta_{d}$ in cm^−1^ eV^1/2^.

It has to be stressed that an indirect transition process has lower probability than the direct transition process, which involves only a photon and an electron. The indirect electronic transition necessarily requires the participation of a phonon. Particularly, for Cu_3_N semiconductor, the gap is an indirect-gap. That gap implies that the absorption coefficient increases gradually with the increase in photon energies until a certain threshold energy. It then increases sharply due to the fact that the energy for optical absorption is determined by the direct transition. We observe in Figure 12 the values of the double-indirect and direct gaps.

The obtained values of the optical resistivity point to the non-conductor nature of the RF-sputtered Cu_3_N thin films under study and relates to the bandgap. Maruyama and Morishita [12], as well as Sahoo et al. [63], have observed the decreasing behavior of the lattice constant with higher deposition pressure, in the case of the reactive RF-sputtering. In addition, Maruyama and Morishita [12] reported that the difference between insulating (non-conducting) and conducting copper nitride was reflected by the differences in the value of the lattice constant, due to the insertion of Cu atoms into the body center of its anti-ReO_2_ crystal structure.

Specifically, the films with a lattice parameter above 3.868 Å were conductors, while otherwise are non-conductors or insulators. Insulating copper nitride layers showed an optical energy gap of approximately 1.3 eV. Sahoo et al. [63], on the other hand, have obtained values of the indirect bandgap up to approximately 1.7 eV. Our observations are indeed in excellent agreement with the above reports, which is to say that the lattice-constant values determined for our RF-sputtered Cu_3_N films are well below 3.868 Å, around 3.81 Å, and are clearly an indirect-gap semiconductor considering our calculated values of the fundamental gap energy, about 1.84 eV. This particular result will be justified in more detail in the next section.

Regarding the Urbach energy $E_{u}$, the estimated high values are listed in Figure 12. According to the literature, copper-nitride semiconductors generally shows values of $E_{u}$ approximately ranging from 100 up to 200meV. The reported Urbach energies of around 300 meV are indeed extremely high, indicating the presence of very significant defect localized states (Urbach-tail-band absorption) [64]. Expanding our discussion, it seems evident that the decrease in the working pressure from 5.0 Pa to 3.5 Pa, up to some extent improves the structural quality of the present copper nitride thin-film material, associated with the decrease in the parameter Eu, from 336 meV down to 263 meV, respectively.

## 9. Phenomenological Absorption Model (PAM)

In the optical absorption edges, corresponding to the strong absorption zone of the transmittance spectra, the absorption coefficient α(E) is related to the optical energy gap $E_{g}^{\mathrm{opt}}$ by following the power-law (Tauc) behavior [65], that is,

(29) $$\alpha E=\beta {\left(E-E_{g}^{\mathrm{opt}}\right)}^{m},$$

where β is an energy-independent constant, and *m* is an exponent related to the electronic nature of the particular bandgap, with values of 3, 2, 3/2, and 1/2, corresponding to indirect forbidden, indirect allowed, direct forbidden, and direct allowed transitions, respectively.

There is generally inconsistent information about the optical properties of the Cu_3_N material. This complexity is probably amplified in nano-materials, as their size and shape can influence the optical properties. These optical properties of the copper nitride compound have been the subject of strong controversy with regard to the bandgap values, as well as the nature of the electronic transitions. The bandgap values are reported to vary between 0.6 and 2.4 eV, and the nature of the bandgap have been reported to be both direct and indirect. From band-structure calculations, on the other hand, one indirect and two direct bandgaps at the M and R points of the reciprocal space, respectively, have been reported following the density functional theory. The indirect bandgap is usually associated with small band-gap values and the direct bandgap transitions are in the visible spectral region, as mentioned above.

To accurately determine the indirect and direct bandgaps, we carried out a least-squares fit of the spectral dependence of the absorption coefficient, α(E), according to the following phenomenological model [9]:

- For $E<E_{1}:\alpha \left(E\right)=\alpha_{0}\exp\left(E/E_{u}\right)$, where $E_{1}$ is the first absorption transition energy, $E_{u}$ is the Urbach energy, and $\alpha_{0}$ is an absorption pre-factor.
- For $E_{1}<E<E_{2}:\alpha E=\beta_{i}{\left(E-E_{g}^{i}\right)}^{2}$, where $E_{2}$ is the corresponding second absorption transition energy, and $E_{g}^{i}$ is the indirect bandgap energy.
- For $E_{2}<E<E_{3}:\alpha E=\beta_{d}\sqrt{E-E_{g}^{d}}$, where $E_{3}$ is the end of the direct bandgap region, and $E_{g}^{d}$ is the direct bandgap energy.

**Figure 13:**
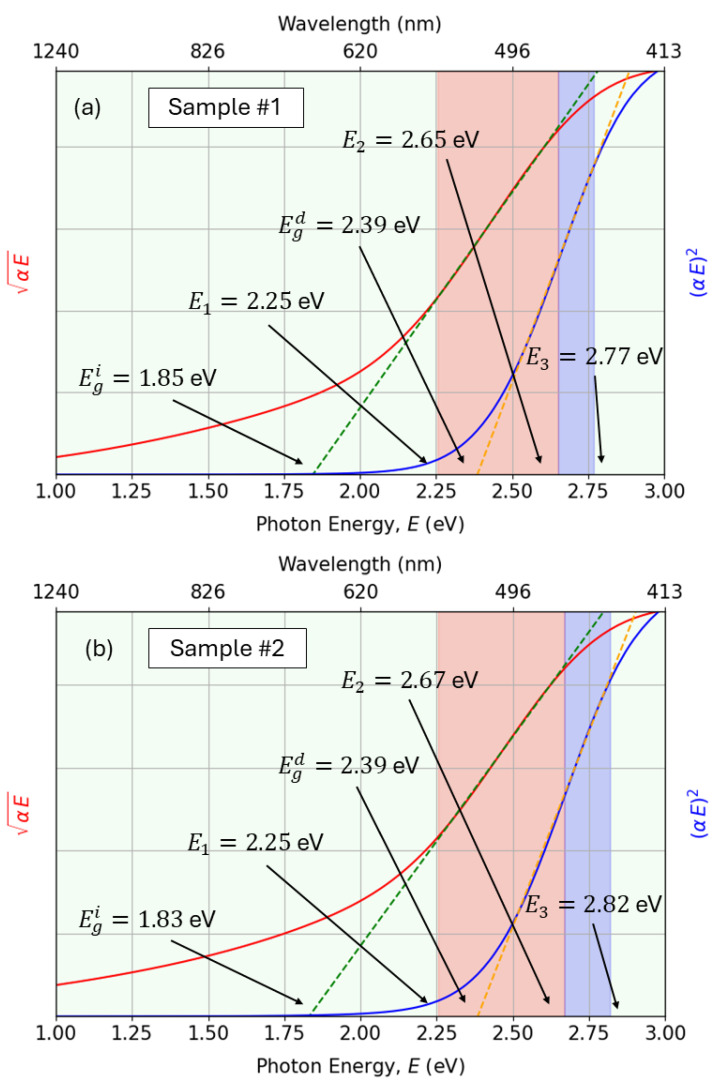
Tauc plots for (**a**) sample #1 and (**b**) sample #2.

The graphs in Figure 13 depict the fit of the experimental data with the adopted PAM described by the previous equations. The red-shaded regions in Figure 13 represent the range of photon energies $\left(E_{1}<E<E_{2}\right)$ associated with the electronic transitions corresponding to the existing indirect gap, $E_{g}^{i}$. Similarly, the blue-shaded regions correspond to the region of the direct gap $E_{g}^{d}$.

The exact parameters $\left(E_{1},E_{2},E_{3}\right)$ were calculated from the Tauc plots (see Figure 13) using, for the first time, an automated optimization approach. In this procedure, a target coefficient of determination ($R^{2}$) was predefined (e.g., $R^{2}=0.998$ to ensure visually and empirically reasonable linear fits). The optimization then searched for the optimal boundaries $\left(E_{1},E_{2}\right)$ such that the indirect transition region produced a linear regression within the desired $R^{2}$ tolerance. A similar procedure was performed for the direct transition region. The optimization was carried out using the global direct-search method, with initial guesses guided by visual inspection of the optical absorption edges. For each candidate interval, a linear regression of either ${\left(\alpha E\right)}^{1/2}$ (indirect) or ${\left(\alpha E\right)}^{2}$ (direct) *versus* photon energy was performed, and the fitting range was iteratively adjusted until the $R^{2}$ exceeded the predefined threshold. The corresponding intercept with the energy axis provided the bandgap values. This novel approach ensures a consistent and reproducible determination of the band edges, reducing the subjectivity inherent to manual selection of the linear fitting regions in Tauc extrapolation plots, as generally performed thus far in the existing literature. Although the optimization yields a unique and well-defined set of parameters corresponding to the optimal fit, slight variations in the predefined $R^{2}$ threshold may lead to marginally different gap values. Such variations, however, remain within a conservative and widely accepted uncertainty range of approximately ±0.05 eV, which mainly reflects the intrinsic limitations of the Tauc-extrapolation method rather than uncertainties in the optimization itself.

In addition to the determination of the transition intervals, the corresponding parameters $\left(E_{u},\alpha_{0},\beta_{i},\beta_{d},E_{g}^{i},E_{g}^{d}\right)$ were obtained by performing linear regressions in the appropriate transformed spaces, namely lnα(E) *versus E* for the Urbach tail, $\sqrt{\alpha E}$ *versus E* for the indirect transition, and ${\left(\alpha E\right)}^{2}$ *versus E* for the direct transition. These fits were evaluated within the automatically optimized energy windows, and the regression quality was quantified by the corresponding $R^{2}$ score. This combined procedure (optimization plus regression) ensures that both the energy boundaries $\left(E_{1},E_{2},E_{3}\right)$ and the other physical parameters are extracted in a consistent, objective, and reproducible manner. The calculated indirect bandgap of 1.84 eV is higher than the optimal range for single-junction solar cells. It should be noted that, according to the Shockley–Queisser detailed balance limit, the maximum theoretical efficiency is about 33% at a bandgap of 1.34 eV, while values between 1.1 eV and 1.6 eV still yield efficiencies above 30%. Nevertheless, the obtained bandgap using the devised automated Tauc-fitting methodology lies within the range typically considered optimal for the top absorber layer in tandem or multi-junction photovoltaic architectures (approximately 1.7–1.9 eV). This is particularly advantageous when combined with silicon, whose bandgap is 1.1 eV. In such configurations, a larger bandgap enables higher open-circuit voltages and efficient harvesting of the high-energy portion of the solar spectrum, while transmitting lower-energy photons to the bottom cell.

## 10. Single-Effective-Oscillator Analysis Below the Bandgap Energy

The fitting of the calculated copper-nitride refractive-index optical dispersion below the bandgap energy (i.e., the transparency spectra region) is performed according to the Wemple–DiDomenico (WDD) single-effective-oscillator model [66], given by

(30) $$n^{2}\left(E\right)=1+\frac{E_{0}\;E_{d}}{E_{0}^{2}-E^{2}},$$

where $E_{0}$ is the energy of the dispersion oscillator, and $E_{d}$ is the so-called dispersion energy.

**Figure 14:**
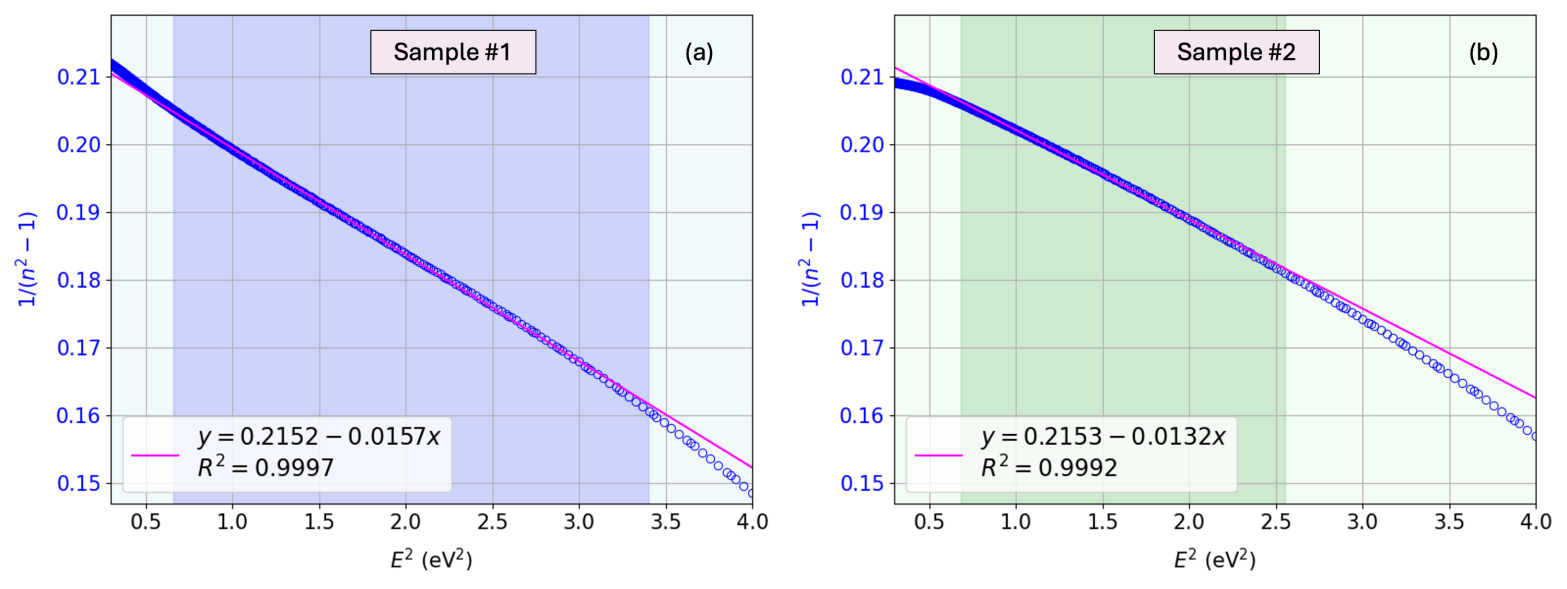
WDD model for (**a**) sample #1 and (**b**) sample #2.

The two Wemple–DiDomenico parameters, $E_{0}$ and $E_{d}$, were found by plotting the refractive-index factor $\left(n^{2}-1\right)$ *versus* $E^{2}$ (see Figure 14). More specifically, these values of $E_{0}$ and $E_{d}$ were estimated from the slope, $-1/(E_{0}\;E_{d})$, and the “incident-photon–energy E=0 intercept”, $E_{0}/E_{d}$, of the Wemple–DiDomenico plot.

The calculated values of $E_{0}$ and $E_{d}$ are presented in Figure 14. The curves displayed in that figure illustrate the general features described by Wemple–DiDomenico, after investigating over 100 crystalline and non-crystalline materials [67]. At long wavelengths (low photon energies), a curvature deviation from linearity is usually observed, due to the contribution of lattice vibrations to the refractive index. At short wavelengths (high photon energies), a negative curvature deviation is very often observed, due to the proximity of the band-edge absorption. However, a sufficiently extended region of linearity is observed to allow for the unambiguous experimental determination of the two parameters $E_{0}$ and $E_{d}$.

The single-oscillator energy, $E_{0}$, is interpreted as a weighted average energy for optical transitions. For the dispersion energy $E_{d}$, an empirical relationship was additionally suggested [67],

(31) $$E_{d}=\beta \;N_{c}\;Z_{a}\;N_{e}\;(\mathrm{in}\;\mathrm{eV}),$$

where β is the empirical constant equal to 0.37±0.04 eV for covalent materials (as in the present case of the Cu_3_N compound), or equal to 0.26±0.03 eV for materials with ionic character. Moreover, $N_{c}$ is the coordination number of the cation nearest neighbor of the anion (copper is the cation in copper nitride, with $N_{c}=2$), $Z_{a}$ is the formal chemical valency of the anion (nitrogen in our binary compound, with $Z_{a}=3$), and $N_{e}$ is the effective number of valence electrons for each nitrogen anion. In the copper nitride compound, it is verified that

(32) $$N_{e}=\frac{\left(3\;\mathrm{valence}\;\mathrm{electrons}\;\mathrm{from}\;3\;\mathrm{Cu}\;\mathrm{cations})+(5\;\mathrm{valence}\;\mathrm{electrons}\;\mathrm{from}\;1\;N\;\mathrm{anion}\right)}{1\;N\;\mathrm{anion}}=8.$$

We are not taking into account the Cu *d* electrons in the previous electron count: in the case of Cu_3_N, a $d^{10}$ core-electron contribution is not expected (unlike Cu halides). Including them would forcibly add 10 more effective valence electrons per Cu cation to $N_{e}$ and would lead to a notable disagreement between experimental and calculated values of $E_{d}$.

With these suggested parameters, Equation (31) yields

$$E_{d}=\beta N_{c}Z_{a}N_{e}=\left(0.37\pm 0.04\right)\times 2\times 3\times 8=17.8\pm 1.9\;\mathrm{eV}.$$

Please note that the ionic-case estimate would have yield $E_{d}=12.5\pm 1.4$ eV for β=0.26±0.03 eV. The experimental values extracted from the Wemple–DiDomenico fits (see Figure 14) are $E_{d}^{\exp}\approx 17.2$ eV for sample #1 and $E_{d}^{\exp}\approx 18.8$ eV for sample #2, which clearly lie within the range predicted by Equation (31). The well-known sensitivity of the RF-sputtered Cu_3_N layers must be taken into account when analyzing the adjustment of the dispersion parameter $E_{d}$.

We also considered an alternate line of reasoning, in which we seek to extract the value of the scale factor β in Equation (31) from the experimental value of $E_{d}$. In that case, β has been found to be equal to $E_{d}^{\exp}/\left(N_{c}Z_{a}N_{e}\right)\approx 0.36$ eV and 0.39 eV for the two representative samples #1 and #2, respectively. These two values are certainly within the range β=0.33–0.41 eV, corresponding to materials where the nature of the atomic bonding is predominantly covalent, thus confirming the initial assumption on the chemical-bonding character in the copper nitride compound.

Furthermore, the refractive index n(0) can be expressed by the following equation:

(33) $$n^{2}\left(0\right)=1+\frac{E_{d}}{E_{0}}.$$

Using the experimental WDD parameters obtained above, namely $\left(E_{0},E_{d}\right)=(3.70\;\mathrm{eV}$, 17.2eV) for sample #1 and (4.04eV,18.8eV) for sample #2, this relation results in n(0)≈2.38 for both specimens (more precisely, n(0)=2.377 and 2.378, respectively, with four significant digits). It can be seen that the values of n(0) for our Cu_3_N specimens are also, in practice, quite coincident with each other.

Moreover, it has been found empirically that $E_{0}=c\;E_{g}^{\mathrm{direct}}$, where the lowest value of the direct bandgap energy, $E_{g}^{\mathrm{direct}}$, is considered in the expression, and *c* is a constant, typically within the range 1.5–2.0. The calculated $E_{0}$ values are consistent with the above proportionality when the direct gaps extracted from the Tauc analysis are used. For sample #1, $E_{0}/E_{g}^{\mathrm{direct}}\;\approx \;1.55$ using $E_{g}^{\mathrm{direct}}\;\approx \;2.39$ eV, and a similar consistency is found for sample #2. On the other hand, this dispersion parameter $E_{0}$ has been found to be between the main two Tauc–Lorentz peaks in the $\epsilon_{2}\left(E\right)$ spectrum (see Figure 10), as expected.

Additionally, there is a well-known trend that lower bandgaps increase with an increasing interatomic bond length, $d_{\mathrm{bond}}$. In the case of the Cu_3_N cubic crystal structure, it has been determined that $d_{\mathrm{bond}}=a/2$, where *a* is the lattice constant. Specifically, a simple power law of the form $E_{0}\propto d_{\mathrm{bond}}^{-s}$ has been reported, where 2<s<3. On the contrary, the dispersion parameter, $E_{d}$, depends on the bond length according to $E_{d}\propto d_{\mathrm{bond}}^{\;s}$. Hence, the variation of the Wemple–DiDomenico dispersion parameters, $E_{0}$ and $E_{d}$, with the particular deposition conditions used in order to grow the Cu_3_N layers, indicate a change in the inter-atomic bond length corresponding to the covalent bonding of copper with nitrogen. The Penn model [68] provides the expression:

(34) $$\epsilon \left(0\right)=n^{2}\left(0\right)=1+\frac{2}{3}\left(\frac{E_{\mathrm{plasmon}}}{E_{\mathrm{Penn}}}\right),$$

where $E_{\mathrm{plasmon}}$ is the valence-electron plasmon energy, and $E_{\mathrm{Penn}}$ is the effective average gap. This latter coefficient is found to be absolutely comparable to the Wemple–DiDomenico average gap, $E_{0}$. The values of the plasmon energy, $E_{\mathrm{plasmon}}$, estimated from Equation (34) are found to be 9.8 eV and 10.7 eV, for the representative Cu_3_N samples #1 and #2, respectively. Moreover, the average bond length, $d_{\mathrm{bond}}$, is related to $E_{\mathrm{Penn}}$ by the semi-empirical expression: $E_{\mathrm{Penn}}\propto d_{\mathrm{bond}}^{-2.5}$. This relationship is consistent with the one previously expressed concerning the relationship between the Wemple–DiDomenico average gap $E_{0}$ and bond length $d_{\mathrm{bond}}$, and hence with the lattice parameter, *a*.

## 11. Conclusions

In this work, we presented a detailed, comprehensive determination of the complex dielectric function of RF-sputtered Cu_3_N thin films on glass substrates. By combining spectroscopic ellipsometry with transmission and reflection measurements, we achieved a synergistic approach that substantially enhances the accuracy of optical characterization.

We employed a multi-oscillator dielectric function that incorporates Tauc, Lorentz, Gaussian, and Drude terms. These oscillators accurately account for the multiple electronic transitions of the polycrystalline Cu_3_N material. Our model provides a consistent description of both direct and indirect bandgaps. In particular, we demonstrated improved bandgap accuracy using a novel phenomenological automated absorption model that constrains the indirect transition in relation to the direct band, mitigating ambiguities very often encountered in previous optical studies of copper nitride films.

It is noteworthy that the optoelectronic performance of our sputtered copper nitride (Cu_3_N) thin films exhibits a relatively moderate yet tunable bandgap, with indirect transitions around 1.84 eV and direct transitions near 2.39 eV. These values are comparable to, but slightly distinct from, those reported for other related binary copper oxides, Cu_2_O (≈2.0–2.6 eV) and CuO (≈1.3–2.2 eV), highlighting the intermediate optical nature of copper nitride within this family of compounds.

Our approach offers a robust framework for determining optical constants and elucidating the fundamental optical response of Cu_3_N. The strategies outlined here can be readily extended to other emerging nitride-based semiconductors, enabling more reliable device design and a deeper understanding of their optoelectronic properties.

## Figures and Tables

**Table 1:** Preparation conditions of the preparation conditions and structural properties for the two representative Cu_3_N samples.

Sample	Sputtering Conditions	Total Pressure (Pa)	Preferred Orientation	Main XRD Peak FWHM	Lattice Constant (Å)	Grain Size, *L* (nm)	Microstrain (%)
#1	Room Temperature	3.5	(100)	0.197°	3.813	41	0.42
#2	N_2_ environment	5.0	(100)	0.187°	3.820	43	0.40

**Table 2:** Results obtained by fitting the experimental spectro-ellipsometric and spectro-photometric data to the proposed multi-oscillator optical model. The precisely determined uniform thickness *d*, non-uniformity wedge $N_{d}$, and roughness $d_{\mathrm{rough}}$ can be compared to the average thicknesses measured by profilometry (${\bar{d}}_{\mathrm{prof}}$) and by SEM (${\bar{d}}_{\mathrm{SEM}}$), albeit possibly at slightly different spots on the film. The n(d) variation column represents the change in refractive index at λ=1500 nm from the front to the backside (adjacent to the substrate) of the film.

Sample	*d* (nm)	$N_{d}$ (nm)	$d_{\mathrm{rough}}$ (nm)	${\bar{d}}_{\mathrm{prof}}$ (nm)	${\bar{d}}_{\mathrm{SEM}}$ (nm)	$\epsilon_{1,\mathrm{offset}}$	n(d) Variation	MSE (%)
#1	133	2.3	8.9	125±5	118±8	1.95	−17%	1.02
#2	109	2.7	7.5	110±6	109±6	1.91	−17%	1.10

**Table 3:** Best-fit parameters for the multiple oscillators of the dielectric function of the two representative Cu_3_N thin films, with their 90% confidence intervals. Parameters marked with (f) are fixed, indicating high confidence in these values.

Oscillator	Sample	A	$E_{0}$ (eV)	Γ (eV)	$E_{g}^{\mathrm{TL}}$ (eV)	$N_{\mathrm{opt}}\;\left(10^{18}\;{\mathrm{cm}}^{-3}\right)$	$\rho_{\mathrm{opt}}\;\left(\Omega \mathrm{cm}\right)$
Gau_1_	#1	0.07(f)	0.19(f)	1.05(f)	N/A	N/A	N/A
Gau_1_	#2	0.57(f)	0.28(f)	2.00 ± 0.01	N/A	N/A	N/A
Gau_2_	#1	3.50 ± 0.01	2.67 ± 0.001	0.63 ± 0.001	–	N/A	N/A
Gau_2_	#2	2.73(f)	2.68 ± 0.0004	0.59 ± 0.001	–	N/A	N/A
TL_1_	#1	8.67(f) eV	3.34(f)	1.49 ± 0.003	0.59 ± 0.002	N/A	N/A
TL_1_	#2	11.3(f) eV	3.28 ± 0.001	1.73 ± 0.002	0.72 ± 0.001	N/A	N/A
TL_2_	#1	91.8(f) eV	4.01 ± 0.01	1.75(f)	3.29 ± 0.002	N/A	N/A
TL_2_	#2	83.5(f) eV	4.02(f)	1.64 ± 0.02	3.34 ± 0.005	N/A	N/A
Pole	#1	30.7 ± 0.2 eV^2^	N/A	6.99(f)	N/A	N/A	N/A
Pole	#2	30.5 ± 0.4 eV^2^	N/A	6.88 ± 0.04	N/A	N/A	N/A
Dru	#1	0.043	1.45	N/A	N/A	6.20(f)	0.25 ± 0.01
Dru	#2	0.014	0.97	N/A	N/A	1.97(f)	0.53 ± 0.04

## Data Availability

The raw data supporting the conclusions of this article will be made available by the authors on request.
